# Performance Verification of CYP2C19 Enzyme Abundance Polymorphism Settings within the Simcyp Simulator v21

**DOI:** 10.3390/metabo12101001

**Published:** 2022-10-20

**Authors:** Caroline Sychterz, Iain Gardner, Manting Chiang, Ramakrishna Rachumallu, Sibylle Neuhoff, Vidya Perera, Samira Merali, Brian J. Schmidt, Lu Gaohua

**Affiliations:** 1 Clinical Pharmacology & Pharmacometrics, Bristol Myers Squibb, Lawrenceville, NJ 08540, USA; 2 Certara UK Ltd., Simcyp Division, Sheffield S1 2BJ, UK

**Keywords:** PBPK, CYP2C19, polymorphism, population, Simcyp Simulator

## Abstract

Physiologically based pharmacokinetic (PBPK) modeling has a number of applications, including assessing drug–drug interactions (DDIs) in polymorphic populations, and should be iteratively refined as science progresses. The Simcyp Simulator is annually updated and version 21 included updates to hepatic and intestinal CYP2C19 enzyme abundance, including addition of intermediate and rapid metabolizer phenotypes and changes to the ultra-rapid metabolizer enzyme abundance, with implications for population clearance and DDI predictions. This work details verification of the updates with sensitive CYP2C19 substrates, omeprazole and lansoprazole, using available clinical data from literature. Multiple assessments were performed, including recovery of areas under the concentration-time curve (AUC) and C_max_ from compiled datasets for each drug, recovery of victim DDI ratios with CYP2C19 and/or CYP3A4 inhibition and recovery of relative exposure between phenotypes. Simulated data were within respective acceptance criteria for >80% of omeprazole AUC values, >70% of lansoprazole AUC and C_max_, >60% of AUC and C_max_ DDI ratios and >80% of exposure ratios between different phenotypes. Recovery of omeprazole C_max_ was lower (>50–70% within 2-fold) and possibly attributed to the variety of formulations used in the clinical dataset. Overall, the results demonstrated that the updated data used to parameterize CYP2C19 phenotypes reasonably described the pharmacokinetics of omeprazole and lansoprazole in genotyped or phenotyped individuals.

## 1. Introduction

Evaluation of the drug–drug interaction (DDI) potential of a drug candidate is a vital part of drug development to properly inform posology in situations where multiple medications may be co-administered. DDI assessment can become even more complex when considering drugs whose elimination is governed by polymorphic drug metabolizing enzymes or transporters, where subgroups in the population, as often defined by allelic variation, can demonstrate different magnitudes of DDI [1]. DDIs are typically evaluated with designated clinical studies to explore the perpetrator or victim potential of a drug candidate; however, regulatory authorities have recognized the utility of computational modeling techniques in this process [2,3]. A recent evaluation of FDA submissions revealed that almost 50% of new drug approvals included physiologically based pharmacokinetic (PBPK) modeling with the majority used for DDI assessment [4].

PBPK modeling is an ideal tool in exploring DDI potential in genotyped or phenotyped individuals as information on the frequency and enzyme abundance associated with different genotypes or phenotypes can be incorporated in the physiological systems information of the simulations. Compiling physiological systems information can be a resource-intensive process requiring periodic updates based upon the latest scientific knowledge and extensive verification to ensure data quality and model performance [5]. However, once obtained, the information can be used in future modeling efforts.

The cytochrome P450 (CYP) enzyme family includes the main enzymes responsible for catalyzing oxidative biotransformation of numerous exogenous substrates [6]. They are located throughout the body; however, higher abundance is noted in organs associated with drug metabolism, namely the liver and intestine [7]. CYP enzymes are of particular importance in drug development because their inhibition, induction, genetic polymorphism or disease-related changes in abundance can have significant impact on the pharmacokinetics of administered drugs [8].

CYP2C19 is a polymorphic drug-metabolizing enzyme belonging to the CYP 2C enzyme family and is the major enzyme responsible for the biotransformation of proton pump inhibitors, such as omeprazole and lansoprazole [9]. Up to 5 enzyme phenotypes have been identified: poor (PM, *2/*2, *3/*3 and *2/*3), intermediate (IM, *1/*2, *1/*3, *2/*17), normal (NM, *1/*1, wild type), rapid (RM, *1/*17) and ultra-rapid (UM, *17/*17) metabolizers [10]. Individuals with the PM phenotype carry two loss-of-function alleles (*2 or *3) resulting in significantly reduced metabolism of CYP2C19 substrates, while those with the UM phenotype carry two increased function alleles (*17) resulting in increased metabolism compared to the wild type (*1). The distribution of these phenotypes differs amongst ethnic groups, with Asian populations demonstrating a higher percentage of PMs compared to Caucasians and African Americans [11].

The Simcyp Simulator v21 (Certara UK Ltd., Sheffield, UK) was released in December 2021, and implemented updates to the adult population characteristics for CYP2C19. These changes included updated values for hepatic and intestinal CYP2C19 enzyme abundance, the addition of IM and RM phenotypes, and changes to the existing UM population (Table 1). The purpose of this work was to verify the newly updated CYP2C19 abundance values in healthy adult populations currently implemented in Simcyp v21 software. To facilitate this investigation, proton pump inhibitors, omeprazole and lansoprazole, were selected as probe substrates because they are largely metabolized by CYP2C19 with minor contributions from CYP3A4 [12]. Both drugs are recognized as sensitive clinical index CYP2C19 substrates for use as victim drugs in clinical DDI studies by the FDA [13], making them ideal probe drugs for assessing CYP2C19 enzyme abundance. The verification was performed in a stepwise manner and included recovery of omeprazole and lansoprazole plasma exposure from compiled datasets, recovery of victim DDI ratios with CYP2C19 and/or CYP3A4 inhibition, and recovery of the relative change in exposure between phenotypes from select studies. The information obtained in this verification was used to assess the reliability of the updated CYP2C19 enzyme abundances described within Simcyp Simulator v21 software for use in prospective PBPK modeling involving CYP2C19 polymorphisms.

## 2. Materials and Methods

### 2.1. Literature Search

A literature search was conducted using the University of Washington Drug Interaction Database [14] and PubMed to identify CYP2C19 pharmacogenomic studies with omeprazole and lansoprazole, including DDI studies.

Plasma concentration-time data, where available, were digitized using MATLAB R2021a digitize2.m code [15]. Only clinical data from healthy adults were included in the analysis; data from pediatrics, elderly or those with severe gastrointestinal conditions (e.g., peptic ulcers) were excluded.

### 2.2. Modeling Strategy

Default settings of Sim-NEurCaucasian, Sim-Chinese and Sim-Japanese virtual populations, as well as the SV-Omeprazole and SV-Lansoprazole compound files within the Simcyp Simulator v21 were used in the verification. Enzyme abundance for each CYP2C19 phenotype is listed in Table 1. Noteworthily, most available clinical studies investigating CYP2C19 polymorphism effects on omeprazole and lansoprazole PK were conducted in Asian subjects, who not only have differences in body height and weight but also characteristically have a higher percentage of PMs [11]. Within the Simcyp Simulator v21 the same enzyme abundance data for Japanese, Chinese and Caucasian populations are assigned while maintaining the different phenotype frequencies between these populations, since PBPK model performance in Japanese and Chinese populations was improved by adjusting CYP2C19 abundance to the same level as Caucasians [16].

Details of omeprazole and lansoprazole parameters used for the simulations are presented in Supplementary Table S1. Briefly, both models incorporate first-order oral absorption. Lansoprazole utilized a full PBPK model while omeprazole incorporated a simplified distribution model which includes liver, small intestine, portal vein and a systemic compartment. Elimination for both drugs utilized intrinsic clearances for CYP2C19 and CYP3A4. Omeprazole is known to inhibit its own metabolism via mechanism-based inhibition of CYP2C19 [17]. Relevant parameters have been incorporated into the SV-Omeprazole model and verified to recover multiple dose omeprazole exposure by the software vendor prior to release. CYP2C19 inhibition parameters for omeprazole were assumed to remain the same across CYP2C19 phenotypes such that any CYP2C19 enzyme abundance differences between phenotypes would be reflected in simulated outcomes.

Model performance verification of CYP2C19 enzyme abundance using omeprazole and lansoprazole consisted of 3 steps, namely:

1. Recovery of area under the concentration-time curve (AUC) and maximum concentration (C_max_) from compiled datasets
2. Recovery of victim DDI ratios with CYP2C19 and/or CYP3A4 inhibition
3. Recovery of the relative change in exposure between phenotypes compared to NM, where available, from select studies

### 2.3. Omeprazole and Lansoprazole PK Prediction in CYP2C19 Phenotypes

Plasma concentration-time data from studies where subjects received a single oral or IV administration were compiled to create a single dataset for each compound per CYP2C19 phenotype [18,19,20,21,22,23,24,25,26,27,28,29,30,31,32,33,34,35,36,37,38,39,40,41,42,43,44,45,46,47,48,49,50,51,52,53,54]. All PK parameters and plasma concentration data were normalized to a 20 mg omeprazole dose or a 30 mg lansoprazole dose assuming dose proportionality around these doses. Theoretically, because SV-Omeprazole and SV-Lansoprazole were described with first-order absorption, no transporter involvement in disposition and linear metabolism (CL_int_), the simulated PK and plasma concentration data can be directly compared to the normalized omeprazole or lansoprazole observations. In practice, lansoprazole demonstrated linear pharmacokinetics with single doses from 15 to 60 mg [55]. Omeprazole single dose exposure was generally proportional between 10 and 40 mg [56], which encompassed the range of doses from the investigated studies. Due to the variety of the study populations from the data collated, simulations were performed using the Sim-NEurCaucasian, Sim-Chinese and Sim-Japanese virtual populations with an age range of 20–50 years and a proportion of 50% female (10 trials with 10 subjects in each trial). Simulated data from each population was compared to the appropriate observed study population, where possible. Due to the absence of specific population data for Korean or Pakistani populations in Simcyp Simulator v21, clinical data was compared to simulated Chinese [57] and Caucasian data, respectively.

Model performance was evaluated by visual inspection of plasma concentration-time curves and recovery of the simulated mean PK parameters (AUC and C_max_) within 2-fold of mean clinical values. Literature references did not specify if means reported were arithmetic or geometric means, therefore arithmetic means were assumed. Because the compiled dataset represented multiple trials and populations, the median and range of the 30 simulated trial means (n = 10 per simulated ethnic group) were plotted against the median and range of the compiled clinical studies to visually assess performance.

### 2.4. Victim DDI Prediction in CYP2C19 Phenotypes

Simulations of DDI studies where omeprazole and lansoprazole were the victim of CYP2C19 and/or CYP3A4 interactions were performed using the same demographic information as the clinical study (i.e., age, sex), if available, otherwise the same settings as described in the previous section were used. Default compound files within the Simcyp Simulator for perpetrator drugs SV-Clarithromycin, SV-Ticlopidine and SV-Fluvoxamine were used. Trial study designs were simulated 10 times with study-matched subject numbers per phenotype to evaluate the range of results from simulations. Model performance was evaluated by comparing simulated mean AUC and C_max_ ratios to clinical results using criteria suggested by Guest et al. [58].

### 2.5. Prediction of CYP2C19 Phenotype Ratios

Simulations were performed as described in Section 2.4. Phenotype ratios were compared to NM, where available. If NM data was not available within a study, IM data was used as the comparator because enzyme abundance was the most similar to NM. Model performance was evaluated as described in Section 2.4 where criteria suggested by Guest et al. [58] was applied.

## 3. Results

### 3.1. Literature Search

Results of the literature search for pharmacogenomic data available for CYP2C19 substrates, omeprazole and lansoprazole, are reported in Supplementary Table S2. The literature search identified 26 studies for omeprazole and 11 studies for lansoprazole administered as single oral or intravenous doses.

S-mephenytoin, another CYP2C19 substrate, was included in the search however only 1 study was identified that measured plasma concentrations across PM, IM and NM CYP2C19 phenotypes [59], while the remaining identified studies measured urinary metabolic ratio and did not report plasma pharmacokinetics (PK). Thus, further evaluation including S-mephenytoin was not conducted.

### 3.2. Omeprazole and Lansoprazole PK Prediction in CYP2C19 Phenotypes

**Figure 1:**
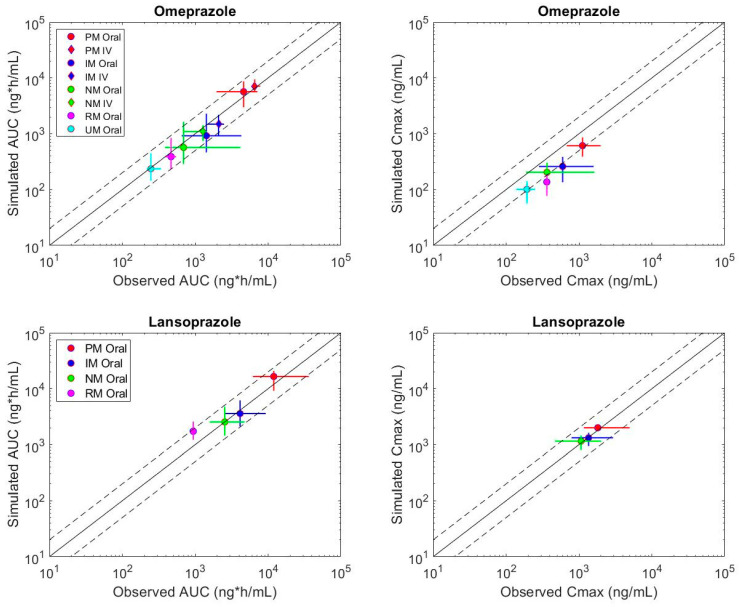
Simulated versus observed median and range of mean AUC and C_max_ values across CYP2C19 phenotypes for omeprazole and lansoprazole. Median of simulated trial means plotted against median of clinical study means. The red, blue, green and magenta vertical lines are range of simulated trial means; horizontal lines are range of clinical study data (see Table 2 for number of studies per phenotype). The solid black line is the line of unity; black dashed lines indicate 2-fold upper and lower acceptance criteria.

The simulated mean AUC for omeprazole recovered >80% of clinical AUC within the 2-fold acceptance criteria per phenotype (Table 2, Figure 1, Supplementary Table S3). Simulated mean C_max_ did not perform as well, recovering approximately 50–70% of the clinical C_max_ within the 2-fold acceptance criteria. Simulated omeprazole C_max_ tended to be under-predicted across phenotypes. As noted in the discussion, different formulations were used in the studies, which may contribute to the observed differences. The simulated mean AUC for lansoprazole recovered >70% and >90% of clinical AUC and C_max_, respectively, within the 2-fold acceptance criteria per phenotype (Table 2, Figure 1, Supplementary Table S3). Simulated and observed mean plasma concentration-time curves for omeprazole and lansoprazole are presented in Supplementary Figure S1 through Supplementary Figure S3.

### 3.3. Victim DDI Prediction in CYP2C19 Phenotypes

Two DDI studies with CYP2C19 phenotyping information for omeprazole were available using either clarithromycin or ticlopidine as a CYP3A4 or CYP2C19 inhibitor, respectively [21,29]. Three DDI studies were available for lansoprazole using either clarithromycin (n = 1) or fluvoxamine (n = 2) as a CYP3A4 or combined CYP3A4/CYP2C19 inhibitor, respectively [34,41,52].

**Figure 2:**
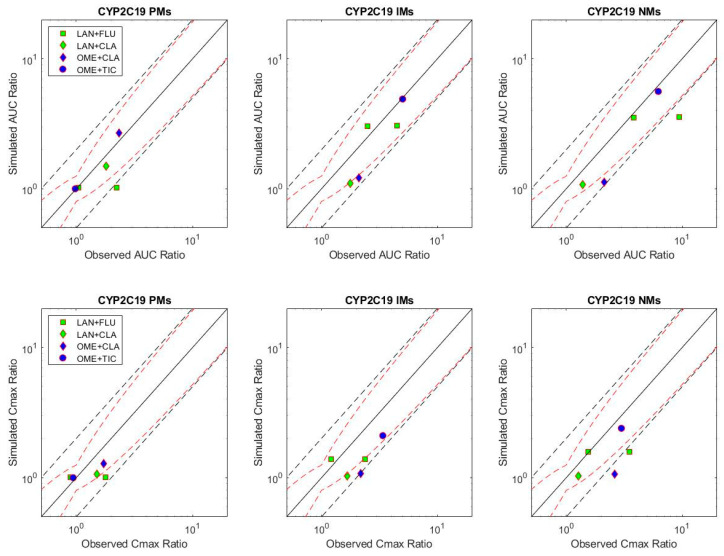
Simulated versus observed mean AUC and C_max_ ratios across CYP2C19 phenotypes for omeprazole and lansoprazole. CLA = clarithromycin; FLU = fluvoxamine; LAN = lansoprazole; OME = omeprazole; TIC = ticlopidine. The solid black line is the line of unity, black dashed lines indicate 2-fold upper and lower acceptance criteria and red dashed lines indicate acceptance criteria suggested by Guest et al. [58].

Mean AUC DDI ratios for 4/5 studies were within the Guest et al. [58] acceptance criteria for CYP2C19 PMs and 3/5 studies for CYP2C19 IMs and NMs (Figure 2). Mean C_max_ DDI ratios for 4/5 studies were within acceptance criteria for CYP2C19 PMs, 2/5 studies for CYP2C19 IMs and 3/5 studies for CYP2C19 NMs. Of the 2 studies investigating DDI between lansoprazole and fluvoxamine, simulated DDI ratios consistently under-predicted data from Miura et al. 2005 [34] while accurately predicting data from Yasuri-Furukori et al., 2004 [52]. The reason for this is unknown as both studies used similar subject groups (age and population) and lansoprazole formulation although the Miura study used a higher dose (60 versus 40 mg).

### 3.4. Prediction of CYP2C19 Phenotype Ratios

**Figure 3:**
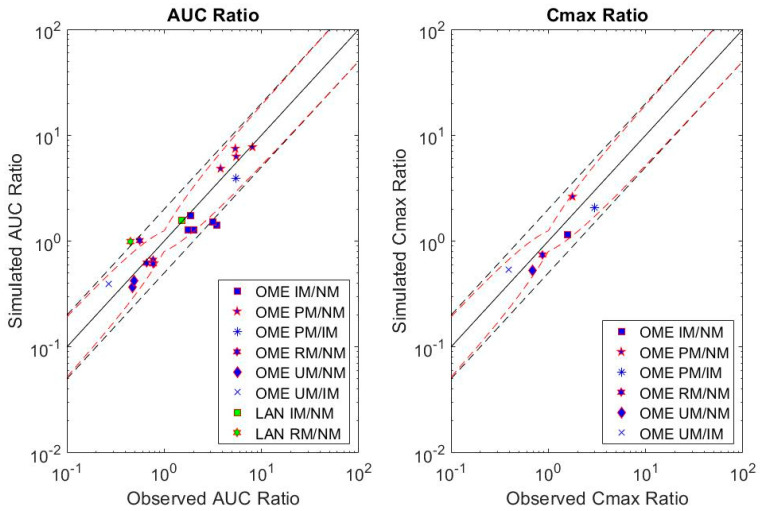
Simulated versus observed mean relative changes in exposure between CYP2C19 phenotypes for select omeprazole (OME) and lansoprazole (LAN) studies [18,19,26,33,40]. The solid black line is the line of unity, black dashed lines indicate 2-fold upper and lower acceptance criteria and red dashed lines indicate acceptance criteria suggested by Guest et al. [58].

Of the studies compiled, five were selected to evaluate phenotype ratios based on their capturing a range of CYP2C19 phenotypes [18,19,26,33,40]. Approximately 80% of simulated mean AUC and 100% of simulated mean C_max_ relative change in exposure between CYP2C19 phenotypes were within the Guest et al. [58] acceptance criteria (Figure 3).

## 4. Discussion

PBPK models have proven to be a useful tool in understanding DDIs to the extent that in some situations, simulations can be used in place of clinical studies [2]. As such, it is important that models remain updated with the newest scientific developments and information. Model reproducibility remains a concern with some of the inconsistency observed attributed to changes in software versions [60]. While proper verification of model performance is necessary for any software update, modelers also need to understand the differences that exist between software versions. PBPK modeling software, Simcyp Simulator, is updated annually and version 21 included updates to CYP2C19 phenotype population data.

S-mephenytoin, omeprazole and lansoprazole are CYP2C19 substrates recommended by the FDA for use in clinical DDI studies [13]. While S-mephenytoin is considered as a sensitive CYP2C19 substrate, it was excluded from this analysis due to scarcity of clinical data with differing CYP2C19 phenotypes. This is likely the reason why only omeprazole and lansoprazole are noted as clinical index substrates for CYP2C19 by the FDA [13]. Using the default settings of healthy volunteer virtual Caucasian, Chinese and Japanese populations, as well as SV-Omeprazole, SV-Lansoprazole and perpetrator drug compound files within the Simcyp Simulator v21, the performance of CYP2C19 enzyme abundance updates was verified.

Verification was carried out in a stepwise manner. First, results from a single simulation were compared to a compiled data set per phenotype for single dose omeprazole or lansoprazole as an assessment of general population performance. Secondly, victim DDI of omeprazole with clarithromycin or ticlopidine and lansoprazole with clarithromycin or fluvoxamine were simulated. Finally, selected studies from the compiled dataset were modeled replicating individual study designs in terms of demographics to assess relative changes in exposure between CYP2C19 phenotypes normalized to NM simulations, where data allowed or to IM subjects if no NM data was available.

Overall, the simulations demonstrated reasonable performance of the updated Simcyp CYP2C19 enzyme abundance information in capturing omeprazole and lansoprazole exposure, DDI effects across CYP2C19 phenotypes and relative change in exposure between phenotypes. Where simulated predictions of omeprazole and lansoprazole exposure were outside of acceptance criteria, the PBPK model had a tendency to underpredict the exposure. Of note, the model under-predicted omeprazole exposure for the two studies that included a Pakistani population regardless of whether clinical data was compared to simulated Caucasian, Japanese or Chinese populations [35,36]. This may reflect unique population characteristics currently lacking in the virtual populations available in Simcyp Simulator v21. Limited data was available for CYP2C19 RM and UM subjects having received either omeprazole or lansoprazole. Further verification of these CYP2C19 phenotypes is warranted as additional data becomes available in the public domain.

Simulated data for lansoprazole was more accurate in recovering clinical C_max_ data than omeprazole. Different formulations of omeprazole were noted between studies, including enteric coated and MUPS (multi-unit pellet system) which are designed to have alternate PK profiles. The omeprazole compound file within v21 of the Simcyp Simulator was designed to recover the exposure of enteric coated omeprazole formulations and it is likely that the C_max_ for other formulations could be more accurately predicted using alternative absorption rate values.

CYP2C19-mediated DDI of omeprazole by ticlopidine was better predicted than CYP3A4-mediated DDI of clarithromycin across CYP2C19 phenotypes which may reflect future opportunities for improvement in either the clarithromycin or omeprazole compound files. Furthermore, CYP2C19 PM enzyme abundance is defined as no enzyme activity and simulation performance may reflect where the compound file models themselves could be further refined. This verification only included two CYP2C19 substrates; additional verification with other CYP2C19 substrates will further inform system parameter performance.

In conclusion, the latest updates to CYP2C19 enzyme abundances within the Simcyp Simulator v21 reasonably recaptured observed data with sensitive CYP2C19 substrates to warrant its use in PBPK simulations for DDI and polymorphism evaluation.

## Figures and Tables

**Table 1:** Updated Hepatic and Intestinal CYP2C19 Enzyme Abundance Implemented in the Simcyp Simulator v21 Compared to v20.

	**Hepatic Abundance (pmol/mg protein) (CV%)**				
**Simcyp**	**PM**	**IM**	**NM**	**RM**	**UM**
V21	0	2.85 (52)	4.4 (52)	7.01 (89)	10.23 (79)
V20	0	NA	4.4 (71)	NA	8.7 (71)
	**Intestinal Abundance (nmol/small intestine) (CV%)**				
**Simcyp**	**PM**	**IM**	**NM**	**RM**	**UM**
V21	0	1.29 (52)	2 (52)	3.18 (89)	4.65 (79)
V20	0	NA	2 (77)	NA	4 (77)

NA = not available.

**Table 2:** Summary of Simulated versus Observed AUC and C_max_ Means Across CYP2C19 Phenotypes for Omeprazole and Lansoprazole.

	No. of Simulated within 2-Fold of Observed/Total No. Data Points				
Omeprazole ^1^	PM	IM	NM	RM	UM
AUC	25/26	22/26	24/27	2/2	3/3
C_max_	12/17	8/16	10/17	0/1	1/2
**Lansoprazole ^1^**					
AUC	7/10	10/11	11/11	1/1	NA
C_max_	9/10	9/10	9/10	NA	NA

NA = Not available, ^1^ Omeprazole includes both oral and IV data; lansoprazole is oral data only.

## Data Availability

Data is contained within the article or supplementary material.

## Supplementary Materials

The following supporting information can be downloaded at: https://www.mdpi.com/article/10.3390/metabo12101001/s1, Figure S1: Simulated and observed mean omeprazole plasma concentration-time data in single oral dose studies per CYP2C19 phenotype; Figure S2: Simulated and observed mean omeprazole plasma concentration-time data in single intravenous dose studies per CYP2C19 phenotype; Figure S3: Simulated and observed mean lansoprazole plasma concentration-time data in single dose studies per CYP2C19 phenotype; Table S1: PBPK model parameters for omeprazole and lansoprazole; Table S2: CYP2C19 literature search results for omeprazole, lansoprazole and S-mephenytoin; Table S3: Simulated versus observed PK parameters for omeprazole and lansoprazole.
