# Supplementary material for: Performance Verification of CYP2C19 Enzyme Abundance Polymorphism Settings within the Simcyp Simulator v21
Source: Metabolites. 2022 Oct 20;12(10):1001. doi: 10.3390/metabo12101001 (PMC9607610; doi:10.3390/metabo12101001)
Supplement: Supplementary file 1 [file metabolites-12-01001-s001.zip › PerformanceVerificationCYP2C19Simcyp_Sychterz_Supp mat v1.pdf]

Performance Verification of CYP2C19 Enzyme Abundance Polymorphism Settings within the Simcyp Simulator v21

Caroline Sychterz, Iain Gardner, Manting Chiang, Ramakrishna Rachumallu, Sibylle Neuhoff, Vidya Perera, Samira Merali, Brian J. Schmidt and Lu Gaohua

Supplementary Materials

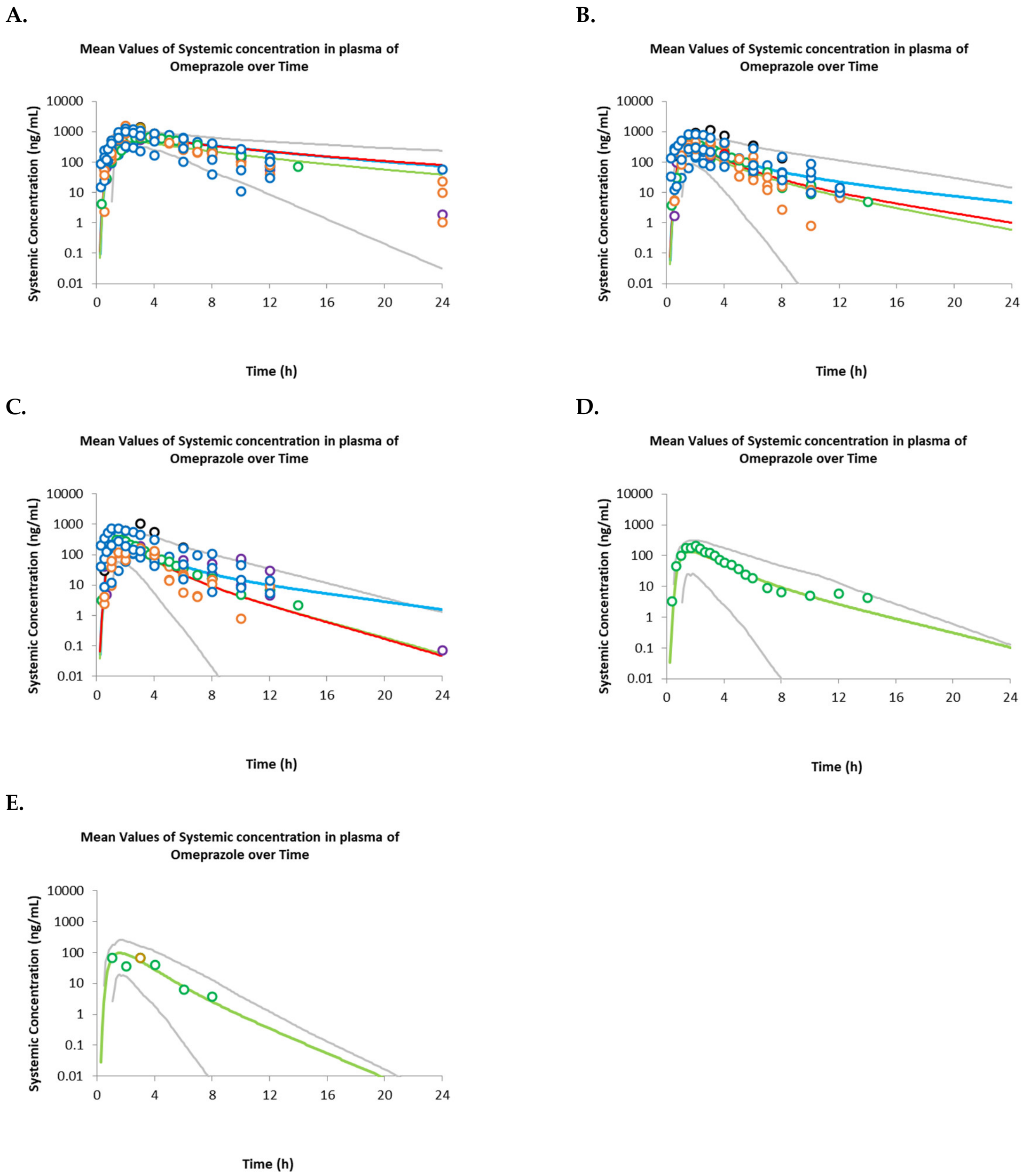

Figure S1. Simulated and observed mean omeprazole plasma concentration-time data in single oral dose studies per CYP2C19 phenotype. A. CYP2C19 poor metabolizers (*2/*2, *3/*3, *2/*3), B. CYP2C19 intermediate metabolizers (*1/*2, *1/*3, *2/*17), C. CYP2C19 normal metabolizers (*1/*1), D. CYP2C19 rapid metabolizers (*1/*17), E. CYP2C19 ultra-rapid metabolizers (*17/*17). Blue line is predicted Chinese mean; red line is predicted Japanese mean; green

line is predicted Caucasian mean; grey lines are 5th and 95th percentiles. Dots are observed data normalized to a 20 mg dose of omeprazole in Chinese (dark blue), Japanese (orange), Caucasian (dark green), Korean (purple), Pakistani (black) and Iranian (gold) subjects. Concentrations at later time points (e.g., 24 h) may reflect imprecision in data extraction from original sources.

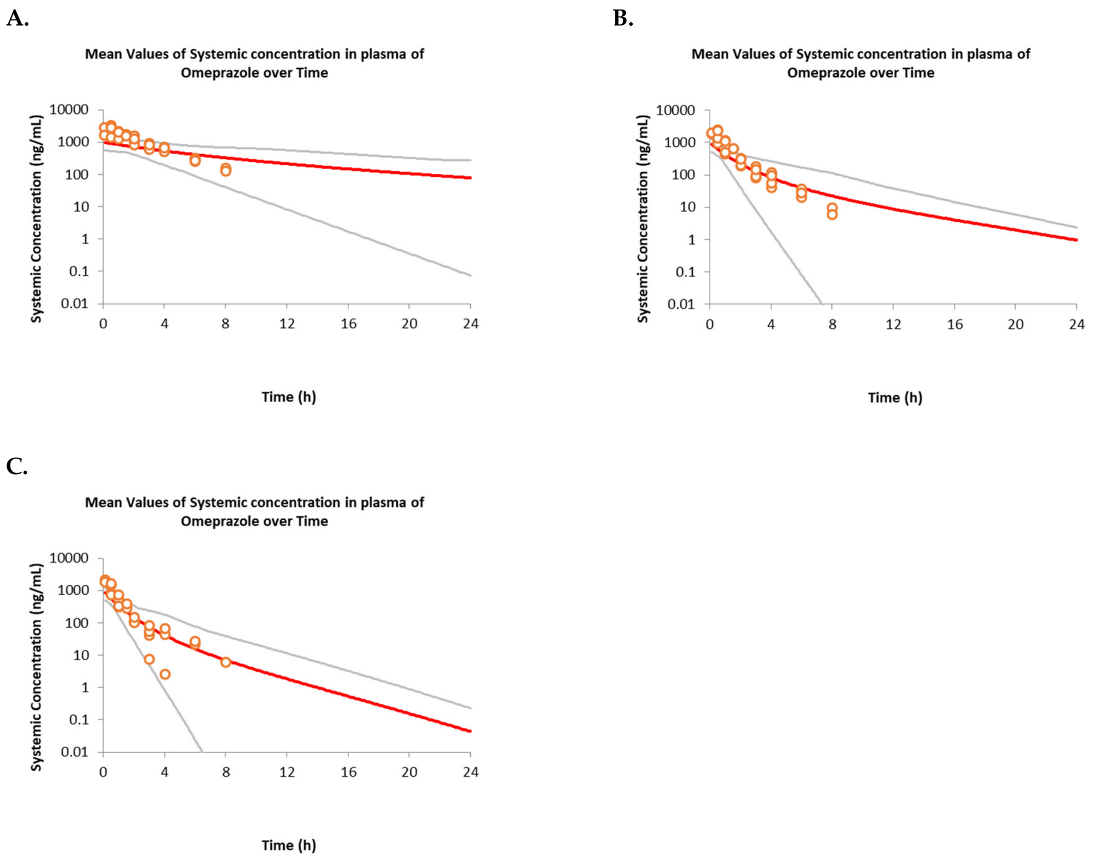

Figure S2. Simulated and observed mean omeprazole plasma concentration-time data in single intravenous dose studies per CYP2C19 phenotype. A. CYP2C19 poor metabolizers (*2/*2, *3/*3, *2/*3), B. CYP2C19 intermediate metabolizers (*1/*2, *1/*3, *2/*17), C. CYP2C19 normal metabolizers (*1/*1). Red line is predicted Japanese mean; grey lines are 5th and 95th percentiles. Dots are observed data normalized to a 20 mg dose of omeprazole in Japanese (orange) subjects.

A.

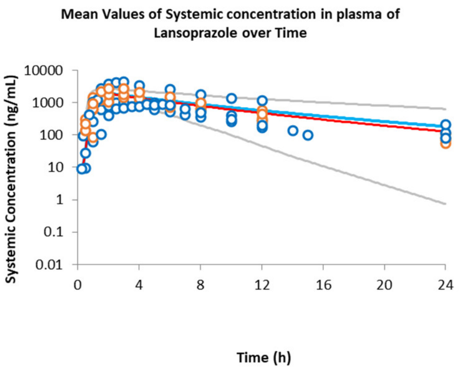

B.

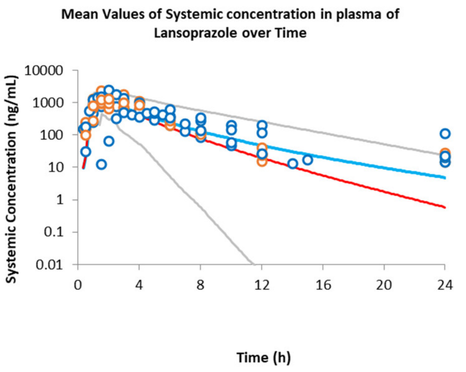

C.

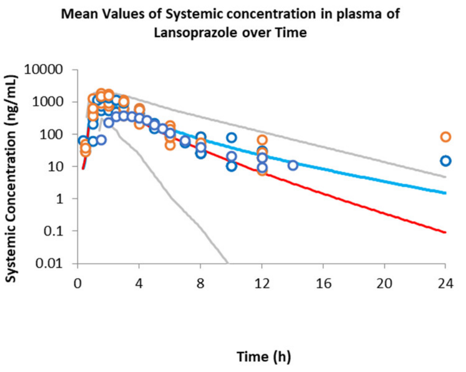

Figure S3. Simulated and observed mean lansoprazole plasma concentration-time data in single dose studies per CYP2C19 phenotype. A. CYP2C19 poor metabolizers (*2/*2, *3/*3, *2/*3), B. CYP2C19 intermediate metabolizers (*1/*2, *1/*3, *2/*17), C. CYP2C19 normal metabolizers (*1/*1). Blue line is predicted Chinese mean; red line is predicted Japanese mean; grey lines are 5th and 95th percentiles. Dots are observed data normalized to a 30 mg dose of lansoprazole in Chinese (dark blue) and Japanese (orange) subjects**.** Concentrations at later time points (e.g., 24 h) may reflect imprecision in data extraction from original sources.

Table S1. PBPK model parameters for omeprazole and lansoprazole. All parameters were default file parameters provided in Simcyp v21 software.

Parameter	Omeprazole	Lansoprazole
Physical chemistry		
Compound type	Ampholyte	Monoprotic base
Molecular weight (g/mol)	345.4	369.4
LogP	2.33	2.8
fu	0.053 (albumin)	0.03 (albumin)
pKa1, pKa2	9.33, 4.31	4.15
B/P	0.59	0.59
Absorption		
Model	First-order	First-order
Peff (x10^{-4} cm/s)	3.25	7.15
fa	0.98	1
ka (1/h)	1.42	3.12
Distribution		
Model	Minimal PBPK	Full PBPK
Kp model	Rodgers with ion membrane permeability	Poulin-Theil/ Berezhkovskiy
Kp scalar	1	0.2
Vss (L/kg)	0.39	0.23
Elimination		
CYP2C19 CLint (μL/min/pmol)	62.6	30.0
CYP3A4 CLint (μL/min/pmol)	0.201	0.107
Additional clearance HLM CLint (μL/min/mg protein)	NA	12
fu,mic	1	1

NA = not applicable
